# Normal Personality, the Dark Triad, Proactive Attitude and Perceived Employability: A Cross-Cultural Study in Belgium, Switzerland and Togo

**DOI:** 10.5334/pb.520

**Published:** 2020-07-22

**Authors:** Kokou A. Atitsogbe, Michel Hansenne, Paboussoum Pari, Jérôme Rossier

**Affiliations:** 1 Institute of Psychology, CePCO, University of Lausanne, CH; 2 Department of Psychology, PsyNCog Research Unit, University of Liège, BE; 3 Department of Applied Psychology, University of Lomé, TG; 4 NCCR-LIVES, University of Lausanne, CH

**Keywords:** personality traits, ZKA-PQ, dark triad, self-perceived employability, proactive attitude, cross-cultural psychology

## Abstract

The current research examined the link between both normal and malevolent personality, proactive attitude, and self-perceived employability across some highly investigated (Belgium, Switzerland) and under-investigated populations of sub-Saharan Africa (e.g., Togo), considering proactive attitude as a potential mediator and self-perceived employability as an outcome. Conducting such a study in contexts which present notable differences in political organization and linguistic diversity, might contribute to enriching the literature on the relationships between personality and self-perceived employability. A sample of 968 participants aged 18 to 85 including 335 Belgians (50% women), 279 Swiss (58.1% women) and 354 Togolese (43.5% women) completed a French version of the Zuckerman-Kuhlman-Aluja Personality Questionnaire (ZKA-PQ/SF), Short Dark Triad (SD3), Proactive Attitude Scale (PAS), and Perceived Employability Scale (PES). All four instruments exhibited metric invariance but did not systematically show scalar invariance across the three countries. ZKA-PQ/SF’s activity and neuroticism and SD3’s narcissism dimensions predicted perceived employability, and these relations were fully or partially mediated by proactive attitude in all cultural contexts. Moreover, perceived employability was predicted by aggressiveness and psychopathy in the Swiss sample and by sensation seeking in both the Swiss and the Belgian samples. Finally, proactive attitude fully mediated between sensation seeking and employability in Belgium and partially between psychopathy and employability in Switzerland. This study illustrates that the link between personality and employability may be mediated by proactive attitude and that these links may be quite robust across cultures.

## Normal Personality, the Dark Triad, Proactive Attitude and Perceived Employability: A Cross-cultural Study in Belgium, Switzerland and Togo

The 21^st^ century has undergone rapid social, economic and technological changes but also crises that have induced uncertainty and instability in numerous aspects of individuals’ lives, such as employability and divorce. For instance, career paths have become less linear, individuals being more likely to experience several transitions (Rossier, 2015). It has been documented that people who perceive themselves as employable are likely to successfully manage career transitions and achieve career success (Rothwell, Herbert, & Rothwell, 2008). Self-perceived employability (SPE) is defined as “the perceived ability to obtain sustainable employment appropriate to one’s qualification level” (Rothwell et al., 2008, p. 2). SPE—considered either as antecedent or consequence—has been highly investigated in career development research during the past decade (Atitsogbe, Mama, Sovet, Pari, & Rossier, 2019). SPE is one conceptualization of employability among others, which places substantial weight on individual characteristics rather than contextual factors (Guilbert et al., 2016). Empirical studies have shown that SPE is associated with several career-related variables, such as well-being indicators (i.e., job satisfaction), organizational outcomes (i.e., work engagement), intentionality (e.g., entrepreneurial intention), motivational variables (e.g., willingness to undertake training), self-regulatory variables (i.e., career adaptability, emotional intelligence), and personal resources (i.e., general self-efficacy), as well as with sociodemographic variables (e.g., professional status) and the type of contract (Atitsogbe et al., 2019; Udayar, Fiori, Thalmayer, & Rossier, 2018; Van Hootegem, Witte, De Cuyper, & Elst, 2018).

### Personality and perceived employability

Thousands of empirical investigations across cultures have made personality a significant and important predictor accounting for several career variables, such as choice or decision-making, engagement, performance, occupational satisfaction (e.g., job satisfaction, burnout), and general processes such as job search behaviors (Nilforooshan & Salimi, 2016; Tokar, Fischer, & Subich, 1998). However, there is very little knowledge about the dispositional influence on SPE, since, to our best knowledge, there is only one published study using the Five Factor Model (FFM), that has formally investigated such a relation (Wille, De Fruyt, & Feys, 2013). The FFM was framed in terms of five superordinate dimensions of normal personality: *neuroticism, extraversion, openness to experience, agreeableness* and *conscientiousness* (McCrae & Costa, 1999). In Wille et al. (2013), when personality and perceived employability were measured longitudinally in a 15-year interval (T1 → T2), they found that SPE was associated negatively with neuroticism, positively with extraversion, and not significantly with conscientiousness. When assessing concurrent associations (T2), they found the above relations to replicate with a change in the association between SPE and conscientiousness, which was significant and positive. Moreover, in one or the other case, openness and agreeableness were positively and negatively related to SPE, respectively. Fugate and Kinicki (2008) proposed a dispositional model that includes work- and career-related traits such as openness to change and proactivity. Although this model may suggest a link between some personality traits and employability, it has not included basic personality traits such as extraversion and neuroticism. In fact, it is important to elucidate the link between both constructs based on an entire personality model (e.g., the Alternative Five Factor Model).

### The Alternative Five Factor Model and career outcomes

To capture and deepen the relation between personality and SPE, it would be necessary to consider beyond the FFM, alternative personality models such as the alternative five-factor model (AFFM; Aluja, et al., 2010; Blanch, Aluja, & Gallart, 2013). In fact, the AFFM includes dimensions such as *aggressiveness, activity* and *sensation seeking* that could be relevant predictors of career outcomes, above and beyond the FFM. In the AFFM, personality is framed in terms of the five dimensions of aggressiveness, activity, extraversion, neuroticism, and sensation seeking. According to the AFFM framework, aggressive individuals are subject to physical and verbal aggression, anger and hostility. Active individuals are described as having work compulsion, being hard-working, capable of carrying on general activities and being restless. Extraversion is associated with characteristics of being positive, warm and socially open. Neurotic people are described as anxious, subject to depression and dependent on others. Sensation seeking is associated with characteristics such as experience seeking, disinhibition, and impulsivity. Past investigations have shown the usefulness of the AFFM dimension of activity to positively predict career engagement and work engagement (e.g., Nilforooshan & Salimi, 2016; Zecca et al., 2015), while sensation seeking was found to negatively contribute to career adaptability (Rossier, Zecca, Stauffer, Maggiori, & Dauwalder, 2012).

### The dark triad and organizational outcomes

The dark triad, also referred to as toxic personality, is conceptualized as a constellation of three malevolent personality traits: *Machiavellianism* (manipulativeness, callous affect, and strategic calculation), *narcissism* (ego reinforcement), and *psychopathy* (deficits in affect and self-control) (Jones & Paulhus, 2014). Some researchers have claimed that the dark triad components are interchangeable (McHoskey, Worzel, & Szyarto, 1998). Positive and relatively high correlations are systematically observed between the three constructs (Furnham, Richards, & Paulhus, 2013). However, according to Paulhus and Williams (2002), these three dimensions seem to have some specific contributions. For instance, a recent meta-analysis has shown that the components of the triad distinctively predict work outcomes such as job performance and counterproductive work behavior (O’Boyle, Forsyth, Banks, & McDaniel, 2012). In particular, Machiavellianism and psychopathy, unlike narcissism, were significantly related to job performance. Moreover, it is worth noting that the predictive effect of Machiavellianism was larger than psychopathy in predicting counterproductive work behavior. Regarding the predictive power of toxic personality compared to bright traits, Scherer, Baysinger, Zolynsky, and LeBreton (2013) have demonstrated that psychopathy, beyond and above the FFM, predicted counterproductive work behavior. More precisely, among all the five FFM traits, only neuroticism significantly predicted the outcome (b = .18) against psychopathy (b = .25). These findings support the usefulness of considering toxic personality and its substantial incremental power in explaining career variables.

### The present study

Given that the context may have an important impact on people’s perceived employability (Guilbert et al., 2016), it would be of interest to study the link between personality and SPE across cultures. For this reason, this study aimed to assess the predictive role of normal and malevolent personality traits on SPE and additionally, the role of the potential mediator of proactive attitude across populations in westernized/industrialized (Belgium, Switzerland) and non-westernized/less industrialized contexts (Togo). Although these three countries share French as an official language, relatively large differences can be observed in their political organization and linguistic diversity. For example, Belgium is a federal state with three main communities based on languages (Flemish, French, and German); Switzerland is a confederation of 26 independent cantons grouped into three linguistic regions (French, German, Italian); and Togo is a republic that includes 40 ethnic languages. These countries may also present significant differences in national culture characteristics, for example in terms of power distance, individualism *versus* collectivism, masculinity *versus* femininity, uncertainty avoidance, long-term *versus* short-term orientation, and indulgence *versus* restraint, among others (Hofstede, Hofstede, & Minkov, 2005). While Belgium and Switzerland may be relatively similar on these dimensions, they differ more from Togo and nearby countries such as Burkina Faso, which, unlike Belgium and Switzerland, is described as collectivist and short-term oriented (Hofstede, 2001; Hofstede et al., 2005). As cultural conditions have been documented to shape individuals’ behaviors (Riordan & Vandenberg, 1994), it would be relevant to investigate the relations between personality (described as stable across cultures) and employability (described as a context-dependent variable) across very different cultural contexts.

*Predicting SPE from AFFM and SD3 traits*. Considering the results of Wille et al. (2013) and the notion that developing a positive interpersonal relationship and initiating active behaviors will favor the development of SPE (see Guilbert et al., 2016 for review), we hypothesized that the AFFM’s aggressiveness (H1) and neuroticism (H2) will be negatively related to SPE, whereas activity (H3), extraversion (H4), and sensation seeking (H5) will be positively related to SPE across countries. We also expect that higher levels in toxic traits such as strategic manipulation and cynicism (Machiavellianism), inflated self-views (e.g., narcissism) and callous social attitudes and impulsive (e.g., psychopathy) would be positively related to one’s perceived ability to obtain sustainable employment. We thus hypothesized that the Dark Triad dimensions will positively relate to SPE across countries (H6).

*The mediating role of proactive attitude*. According to Potgieter and Coetzee (2013), proactivity refers to “accepting responsibility for one’s decisions, setting challenging targets for oneself and identifying opportunities before other people do” (p. 3). Based on this definition, proactivity could be a characteristic adaptation in the terminology of the FFM. In fact, several models consider that the link between personality dimensions and outcomes may be mediated by characteristic adaptations, such as attitudes, goals, plans, self-image, strategies, values or personal strivings, of the FFM traits (McAdams & Pals, 2006; McCrae & Costa, 1999) or regulation processes (Rossier, 2015). A recent meta-analysis has shown that proactivity is linked to the FFM extraversion, openness, and conscientiousness, to organizational variables such as job performance and to individual variables such as work experience (Thomas, Whitman, & Viswesvaran, 2010). Proactivity has also been asserted to positively contribute to employability (Fugate et al., 2004). Consequently, proactive attitude may relate to both personality and SPE and should mediate this relation (H7).

## Method

### Participants

A convenience sample of 968 French-speaking participants from three countries participated in this study: Belgium (*n* = 335; 34.6%), Switzerland (*n* = 279; 28.8%) and Togo (*n* = 354; 36.6%). Belgian participants were aged 18 to 85 (*M* = 45.15; *SD* = 17.10) and 49.9% women; Swiss participants were 18 to 83 (*M* = 39.77; *SD* = 16.15) and 58.1% women, and Togolese participants were 18 to 64 (*M* = 29.05; *SD* = 9.75) and 43.5% women. The present study was part of a larger multicultural validation study of the shortened Zuckerman-Kuhlman-Aluja Personality Questionnaire (ZKA-PQ/SF) across 18 cultures (Aluja et al., 2019). More precisely, two additional instruments, assessing SPE and proactive attitude, were included in the research protocol among these three countries. All participants volunteered for the study.

### Translations

Three members of our research team at the University of Lausanne, the first and last authors helped by a third team member, translated the Short Dark Triad (SD3; Jones & Paulhus, 2014) into French. This translation was back-translated by another team member and reviewed by the SD3 authors. Items were revised upon agreement between SD3 authors and the authors of this French translation (Appendix A). Furthermore, the Proactive Attitude Scale (Schmitz & Schwarzer, 1999) was translated into French by the same team (Appendix B), following the same procedure without a review of the back-translation by the original authors, although they were allowed to compare the translated version with the original version.

### Instruments

#### The ZKA-PQ/SF

Personality dimensions were assessed using the French version of the Zuckerman-Kuhlman-Aluja Personality Questionnaire (ZKA-PQ/SF; Aluja et al., 2019). This instrument consists of 80 items equally divided into five scales (16 items per scale). Participants were asked to indicate the extent to which each item describes some ways they act and think in certain circumstances using a four-point Likert-type scale ranging from *disagree strongly* (1) to *agree strongly* (4). Internal consistency of each scale was assessed by means of Cronbach’s alpha. We obtained Cronbach’s alpha values of .84, .88, and .81 for aggressiveness, .83, .80, and .72 for activity, .81, .80, and .73 for extraversion, .87, .89, and .83 for neuroticism, and .79, .77 and .58 for sensation seeking in the Belgian, Swiss, and Togolese samples, respectively. All reliability scores were in the acceptable range except the value for the sensation seeking scale in Togo, which was lower.

#### Short Dark Triad

Malevolent personality traits were assessed using the Short Dark Triad (SD3). It consists of 27 items equally divided into three scales (9 items per scale): Machiavellianism, narcissism and psychopathy. Items were rated on a 5-point scale from *strongly disagree* (1) to *strongly agree* (5). Jones and Paulhus (2014) found Cronbach’s alphas of .74, .68 and .72 for Machiavellianism, narcissism and psychopathy, respectively, in a combined Canadian-American country sample. The alpha reliability scores were .72, .73, and .63 for Machiavellianism, .59, .64 and .57 for narcissism, and .71, .67 and .51 for psychopathy in the Belgian, Swiss, and Togolese samples, respectively. Overall, the range of reliability scores in the Belgian and Swiss samples were similar to those found across the original sample (Jones & Paulhus, 2014) and were lower in the Togolese sample.

#### Proactive Attitude Scale

Proactive attitude was assessed using our French-translated version (Appendix B) of the Proactive Attitude Scale (PAS; Schmitz & Schwarzer, 1999). This eight-item measure assesses the participants’ agency regarding a broad range of life situations. Items were rated on a 4-point scale from *not at all true* (1) to *exactly true* (4). Cronbach’s alpha values were .81, .77 and .68 in the Belgian, Swiss, and Togolese samples, respectively.

#### Perceived Employability Scale

Self-perception of employability was assessed using a shortened and adapted version of the Perceived Employability Scale (PES; Praskova, Creed, & Hood, 2015). This ten-item measure evaluates respondents’ perceptions of their capability to find a job and stay in the labor market. It contains items such as “People with the same career choice as me are highly valued”. Items were rated on a 5-point scale from *strongly disagree* (1) to *strongly agree* (5). Cronbach’s alphas were .81, .80 and .71 in the samples from Belgium, Switzerland, and Togo, respectively.

### Procedure

In Belgium and Switzerland, data collection was organized at the University of Liège and the University of Lausanne by the co-authors affiliated to these institutions, respectively. The researchers benefited from the help of undergraduate students in a research practicum. Each student collected surveys from eight adult, volunteer participants in the French-speaking parts of Belgium or Switzerland, using the snowball method. The students received course credit for this effort. In Togo, data collection was planned at the University of Lomé by the first and the last authors. The snowball method was used to collect data from adult volunteers in Lomé. Each participant was compensated with prepaid phone credit of 2000 XOF (local currency equivalent to approximately 3 euros). The Belgian, Swiss and Togolese samples consisted of people with a sufficient knowledge of French to understand the survey well.

### Analyses

Descriptive statistics including means, standard deviations, skewness (*S*), kurtosis (*K*), Cronbach’s alphas and bivariate correlations were computed using SPSS 25. The normality of the data was assessed based on Shapiro-Wilk test with expected *p*-values above .05 (Shapiro & Wilk, 1965; Razali & Wah, 2011), and the skewness and kurtosis Z-values (calculated by dividing *S* and *K* values by their respective standard error), which should range from –1.96 to +1.96.

Confirmatory factor analyses (CFAs) were conducted for each instrument considering the overall data using AMOS 25.0 with maximum likelihood estimation. Fit indices were analyzed for each scale, and the models were adjusted if necessary. The following fit indices were considered: χ^2^ per degree of freedom (χ^2^/*df*), comparative fit index (CFI), Tucker-Lewis index (TLI) and root mean square error of approximation (RMSEA). Values of χ^2^/*df* < 3, CFI and TLI ≥ .90 (Byrne, 2010), and RMSEA ≤ .08 or .05 (Hu & Bentler, 1999) were considered indicative of an acceptable or good fit. Measurement invariance across countries was then separately assessed for each scale by means of multigroup CFAs (see Rossier et al., 2016). Three levels of invariance across countries—configural, metric and scalar—were considered. Cross-cultural methodologists have recommended testing for invariance, the metric being a prerequisite for meaningful cross-group comparisons, and scalar invariance being compulsory for mean score comparison across cultures (Van de Vijver & Leung, 2001). For metric and scalar invariance, criteria of ΔCFI .01 (Cheung & Rensvold, 2002) and ΔRMSEA .015 (Chen, 2007) were considered.

Two separate multiple-group path analyses considering the ZKA-PQ/SF and the SD3 were conducted to identify personality dimensions that significantly contributed to SPE scores across countries, controlling for age and sex. Dimensions of the ZKA-PQ/SF and SD3 that produced significant paths to SPE in at least one country were considered for two separate mediation models, considering proactive attitude as a mediator and SPE as an outcome. Tests of mediation were performed in structural equation modeling using a multiple group approach.

## Results

### Descriptive statistics

Descriptive statistics for all variables are presented in Table 1. Scores of the AFFM aggressiveness, activity, extraversion, neuroticism, and sensation seeking exhibited Shapiro-Wilk’s *p* of .000, .269, .303, .243, and .169 for Belgium, .000, .198, .123, .119, and .090 for Switzerland, and .002, .052, .068, .325, and .058 for Togo, respectively. As observed, only FFM aggressiveness scores were not normally distributed across countries (*p* below .05). Regarding Machiavellianism, narcissism, psychopathy and proactive attitudes, Shapiro-Wilk’s *p* were of .079, .007, .000, and .000 for Belgium, .019, .009, .000, and .000 for Switzerland, and .075, .003, .016, and .000 for Togo, respectively. As can be seen, among those scales, only Machiavellianism scores in the Belgian and Swiss samples were normally distributed. Some authors argued that scores can still be considered if departure from normality meets these standards: skewness and kurtosis Z-values ranging from –1.96 to +1.96 (Razali & Wah, 2011). Given that all *S* and *K* Z-values ranged from –0.69 to +1.78 for the scales that presented a significant Shapiro-Wilk’s *p*, all can be considered for multivariate tests. Overall, most Cronbach’s alphas ranged from acceptable to good for all scales. Correlations between all variables per country and for the total sample are reported in Table 2. Regarding the total sample, SPE related significantly and positively to proactive attitude, personality dimensions of narcissism, activity, sensation seeking, extraversion and Machiavellianism and negatively to neuroticism and aggressiveness. The correlation with psychopathy was nonsignificant. These patterns replicated at the country level with few differences. For instance, nonsignificant correlations were found between SPE and aggressiveness (Belgium) and Machiavellianism (in each of the three countries), whereas a significant correlation was found with psychopathy (Switzerland). With respect to the overall sample, proactive attitude related significantly and positively to narcissism, activity, extraversion and sensation seeking and negatively to neuroticism and aggressiveness. The correlations with Machiavellianism and psychopathy were nonsignificant. These patterns were similar at the county level with the difference that nonsignificant correlations were found regarding aggressiveness (Belgium) and sensation seeking (Switzerland and Togo).

**Table 1:** Means, Standard Deviations, Skewness, Kurtosis and Internal Reliabilities for all Variables by Country.

	Belgium (*n* = 335)					Switzerland (*n* = 279)					Togo (*n* = 354)				

	*M*	*SD*	*S*	*K*	α	*M*	*SD*	*S*	*K*	α	*M*	*SD*	*S*	*K*	α

ZKA-PQ/SF															
Aggressiveness	34.08	6.82	0.50	0.08	.84	34.13	7.98	0.37	–0.17	.88	33.30	7.49	0.37	–0.17	.81
Activity	40.71	6.65	0.09	0.22	.83	40.72	6.30	–0.15	–0.18	.80	45.03	5.64	–0.15	–0.18	.72
Extraversion	47.18	5.98	–0.12	–0.14	.81	48.07	5.97	–0.01	–0.14	.80	47.37	5.91	–0.10	–0.14	.73
Neuroticism	35.76	7.48	0.17	–0.10	.87	35.35	8.15	0.12	–0.08	.89	36.27	7.67	0.12	–0.08	.83
Sensation seeking	40.26	6.87	–0.03	–0.28	.79	40.53	6.84	0.20	0.46	.77	41.58	5.21	0.20	0.46	.58
SD3															
Machiavellianism	26.24	4.86	0.18	–0.01	.72	24.64	5.30	–0.18	0.02	.73	39.22	4.91	–0.18	0.02	.63
Narcissism	23.53	4.09	–0.28	0.30	.59	24.02	4.71	–0.12	0.44	.64	28.46	4.31	–0.12	0.44	57
Psychopathy	18.64	4.91	0.55	0.69	.71	18.18	4.92	0.26	0.21	.67	19.57	4.42	0.26	0.21	.51
Proactive Attitude	3.27	0.46	–0.48	–0.19	.81	3.36	0.43	–0.80	0.35	.77	3.52	0.39	–0.80	0.35	.68
Perceived Employability	3.44	0.64	–0.49	0.67	.81	3.62	0.64	–0.33	–0.08	.80	3.93	0.56	–0.33	–0.08	.71

**Table 2:** Intercorrelations among all variables for Belgium, Switzerland, Togo, and the Total Sample.

Scale	Belgium and Togo										Switzerland and Total sample									

	AG	AC	EX	NE	SS	MA	NA	PS	PA	PE	AG	AC	EX	NE	SS	MA	NA	PS	PA	PE

Aggressiveness (AG)		**.09**	**.01**	.17	.21	.28	.20	.47	**<.01**	**.06**		**.04**	**–.06**	.31	.17	.38	.16	.50	–.14	–.22
Activity (AC)	**.02**		.20	**–.07**	.18	**–.08**	.13	**–.02**	.33	.27	**.03**		**.06**	**–.07**	**.04**	**.10**	.24	–.03	.20	.29
Extraversion (EX)	–.16	.27		–.30	.28	**–.03**	.28	**.03**	.33	.19	–.07	.17		–.28	.22	–.11	.27	–.14	.35	.21
Neuroticism (NE)	.51	**–.02**	–.38		**–.06**	.17	–.20	**.01**	–.23	–.23	.34	**–.04**	–.32		**–.10**	**.11**	–.32	**.10**	–.29	–.31
Sensation seeking (SS)	.20	.31	.14	.21		.14	.37	.33	.29	.32	.19	.19	.22	**.01**		.18	.38	.36	**.09**	.16
Machiavellianism (MA)	.22	.12	**–.07**	.26	.27		.29	.48	**–.06**	**.06**	.25	.14	–.08	.19	.21		.35	.50	**–.08**	**–.02**
Narcissism (NA)	.12	.29	.22	**–.01**	.23	.27		.35	.26	.31	.11	.33	.22	–.13	.33	.40		.40	.27	.24
Psychopathy (PS)	.43	**.08**	–.08	.25	.26	.35	.18		–.20	.15	.45	**.04**	**–.06**	.12	.32	.45	.32		**–.08**	–.12
Proactive Attitude (PA)	–.16	.19	**.10**	–.21	**.08**	**.06**	.23	**–.04**		.46	–.12	.30	.25	–.23	.18	**.04**	.32	**–.02**		.51
Self-Perceived Empl. (PE)	**–.10**	.26	.17	–.15	.13	**.07**	.33	**.02**	.50		–.10	.34	.18	–.20	.23	.12	.39	**.06**	.52	

*Note*: Empl. = Employability; AG, AC, EX, NE, and SS are dimensions of the ZKA-PQ/SF; MA, NA, and PS are those of the SD3. Correlations on the left side are above the diagonal for Belgium and below the diagonal for Togo. Correlations on the right side are above the diagonal for Switzerland and below the diagonal for the total sample; nonsignificant correlations are in bold; *p* < .05 for r < .14 and *p* < .01 for r ≥ .14.

### Confirmatory factor analyses and measurement invariance

CFAs considering the overall sample but the instruments separately usually yielded poor initial fit indices: χ^2^/*df* = 9.23, CFI = .750, TLI = .703 and RMSEA = .092 for the ZKA-PQ/SF, χ^2^/*df* = 5.58, CFI = .939, TLI = .909 and RMSEA = .069 for the SD3, χ^2^/*df* = 7.68, CFI = .904, TLI = .865 and RMSEA = .083 for the PAS, and χ^2^/*df* = 22.72, CFI = .700, TLI = .615 and RMSEA = .150 for the PES. In fact, several researchers have highlighted that most personality inventories usually exhibit lower fit indices when all dimensions are considered simultaneously (i.e., Rossier et al., 2016), which could explain the poor fit observed for both the ZKA-PQ/SF and SD3. Moreover, it has been argued that item-level models, which were considered for both PAS and PES, usually result in large modification indices and poor fit (Thompson & Melancon, 1996). The models were adjusted by covarying error terms that had modification indices above 40 for the ZKA-PQ/SF (we also considered two cross-loadings here; the hostility item on neuroticism and the exhibitionism item on sensation seeking), 30 for the SD3, and 20 for both PAS and PES. This significantly improved the fit indices: χ^2^/*df* = 6.03, CFI = .854, TLI = .819 and RMSEA = .072 for the ZKA-PQ/SF, χ^2^/*df* = 4.25, CFI = .959, TLI = .935 and RMSEA = .058 for the SD3, χ^2^/*df* = 5.14, CFI = .946, TLI = .917 and RMSEA = .065 for the PAS, and χ^2^/*df* = 3.19, CFI = .976, TLI = .961 and RMSEA = .048 for the PES. With the exception of χ^2^/*df*, which is sensitive to sample size, fit indices for the instruments, except for those of the ZKA-PQ/SF, met the statistical requirements.

To test the applicability of each instrument to our three country samples, the above adjusted models were used in four separate multigroup CFAs: χ^2^/*df* = 2.70, CFI = .862, TLI = .829 and RMSEA = .042 for the ZKA-PQ/SF, χ^2^/*df* = 2.41, CFI = .939, TLI = .904 and RMSEA = .038 for the SD3, χ^2^/*df* = 2.12, CFI = .958, TLI = .939 and RMSEA = .034 for the PAS, and χ^2^/*df* = 2.58, CFI = .947, TLI = .915 and RMSEA = .041 for the PES. The configural, metric and scalar invariance were tested to evaluate if the participants of the three countries interpreted the measures in a conceptually similar way. Results are summarized in Table 3. SD3 and PES clearly achieved the three levels of invariance. The PAS nearly reached scalar invariance, but not the ZKA-PQ/SF that exhibited fit indices around threshold or below acceptable standards, when all dimensions were considered together. In fact, Aluja et al. (2019) have demonstrated that this instrument could easily exhibit metric invariance across cultures (including our three countries) but not scalar if its factors were tested independently. Overall, our four scales were fully or nearly metric invariant, allowing the study of interrelations between these instruments across groups.

**Table 3:** Measurement Invariance across the Three Countries for Personality Dimensions, Employability and Proactive Attitude Scales.

Dimensions/scales	χ^2^	*dƒ*	χ^2^/*d*ƒ	*p*	CFI	TLI	RMSEA	Δχ^2^	Δ*d*ƒ	*p*	ΔCFI	ΔTLI	ΔRMSEA

ZKA-PQ/SF (MI ≥ 40)													
Configural Invariance	1239.50	459	2.70	<.001	.862	.829	.042						
Metric Invariance	1349.14	499	2.70	<.001	.850	.828	.042	109.64	40	<.001	.012	.001	<.001
Invariance	1491.29	529	2.82	<.001	.830	.817	.043	142.15	30	<.001	.020	.011	.001
SD3 (MI ≥ 30)													
Configural Invariance	166.46	69	2.41	<.001	.939	.904	.038						
Metric Invariance	189.93	81	2.35	<.001	.931	.908	.037	23.46	12	.024	.008	.004	.001
Scalar Invariance	210.85	93	2.27	<.001	.926	.914	.036	20.92	12	.052	.005	.006	.001
PAS (MI ≥ 20)													
Configural Invariance	114.33	54	2.12	<.001	.958	.934	.034						
Metric Invariance	144.60	68	2.13	<.001	.946	.933	.034	30.27	14	<.001	.012	.001	<.001
Scalar Invariance	164.88	70	2.35	<.001	.933	.920	.037	20.28	2	<.001	.013	.013	.003
PES (MI ≥ 20)													
Configural Invariance	217.02	84	2.58	<.001	.947	.915	.041						
Metric Invariance	235.33	102	2.31	<.001	.947	.930	.037	18.30	18	.435	<.001	.015	.004
Scalar Invariance	242.42	104	2.33	<.001	.945	.928	.037	7.10	2	.029	.002	.002	<.001

*Note*: MI = Modification indices.

### Personality and self-perceived employability in each country

Path analyses revealed that the model with the ZKA-PQ/SF explained 19%, 20% and 16% of the variance in SPE for the samples from Belgium, Switzerland and Togo, respectively. Regarding age and sex, which were controlled, age significantly impacted SPE for Belgian (b = –.19, *p* < .01) and Togolese (b = –.21, *p* < .001) samples. Moreover, sex significantly impacted SPE for those in Togo only (b = –.11, *p* < .05), in favor of women. Only activity exhibited a strong positive effect on SPE in the three samples (Belgium, b = .27; Switzerland, b = .28; Togo, b = .24; all *p* < .001), followed by a negative effect of neuroticism (Belgium, b = –.21, *p* < .001; Switzerland, b = –.19 *p* < .01; Togo, b = –.21, *p* < .001) and aggressiveness (Switzerland, b = –.19 *p* < .001), and a positive effect of sensation seeking (Belgium, b = .19, *p* < .001; Switzerland, b = .19 *p* < .001). The model with SD3 explained 13% of the variance in SPE for the samples from Belgium and Switzerland and 12% for the samples from Togo. Of the controlled variables, only age significantly impacted SPE, which occurred in the Belgian sample (b = –.17, *p* < .01). Only narcissism exhibited a strong positive effect on SPE in the three samples (Belgium, b = .29; Switzerland, b = .33; Togo, b = .33; all *p* < .001), followed by psychopathy with a significant negative effect only in the Swiss sample (b = –.27; *p* < .001).

### Proactive attitudes as a mediator

**Figure 1:**
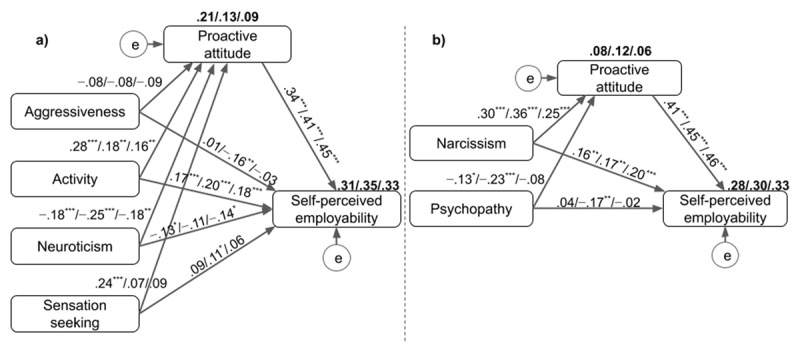
The mediation models showing direct and indirect relations between personality dimensions and perceived employability through proactive attitude for the Belgian, Swiss and Togolese samples, respectively; age and sex were controlled; the percentage of variance explained are in bold; covariation among personality dimensions are not reported (* *p* < .05; ** *p* < .01; *** *p* < .001).

As shown in Figure 1, extraversion and Machiavellianism, which failed to predict SPE in previous analyses, were not included in the mediation model (a) and model (b), which considered the ZKA-PQ/SF and the SD3. The two models fit the overall data well, considering adjustments based on the modification indices above 20. Fit indices for model (a) were χ^2^/*df* = 4.69, CFI = .997, TLI = .905 and RMSEA = .062, and those for model (b) were χ^2^/*df* = .63, CFI = 1.00, TLI = 1.00 and RMSEA = .00. The multigroup mediation tests using these models and 5,000 bootstrap samples with 95% bias-corrected confidence intervals provided overall good fit indices for both model (a), χ^2^/*df* = 2.52, CFI = .996, TLI = .880 and RMSEA = .040, and model (b), χ^2^/*df* = 1.32, CFI = .997, TLI = .978 and RMSEA = .018.

Mediation analyses were based on Shrout and Bolger (2002) recommendations. According to these authors and as reviewed by Nilforooshan and Salimi (2016), a full mediation is observed when the following three conditions are met simultaneously: a significant total effect of the predictor on the criterion (1), a significant indirect effect of the predictor on the criterion via the mediator (2) and a nonsignificant direct effect of the predictor on the criterion after having introduced the mediator in the model (3). A partial mediation is observed when the two last conditions are met simultaneously.

In model (a), the total, indirect and direct effects of ZKA-PQ/SF dimensions on SPE through proactive attitude were tested. As seen in Table 4, the indirect and direct effects of activity on SPE across countries were significant, suggesting that proactive attitude partially mediated between activity and SPE across cultural contexts. Moreover, partial mediation was observed with neuroticism in the Belgian and Togolese samples, while full mediation was observed in the Swiss sample as the direct effect of neuroticism on SPE became nonsignificant. The indirect effect of neuroticism on SPE in this full mediation was particularly strong (b = –.10, *p* < .001). Furthermore, proactive attitude fully mediated between sensation seeking and SPE in the Belgian sample only, and no mediation was observed in the two other samples. Finally, no mediation was observed in the relation involving aggressiveness in any of the three samples.

**Table 4:** Standardized estimates of the paths from the ZKA-PQ and the SD3 dimensions to perceived employability through proactive attitude.

	Belgium (*n* = 335)				Switzerland (*n* = 279)				Togo (*n* = 354)			

	*STE*	*SIE*	*SDE*	Result	*STE*	*SIE*	*SDE*	Result	*STE*	*SIE*	*SDE*	Result

ZKA-PQ												
Aggressiveness	–.02	–.03	.01	No med	–.19**	–.03	–.16**	No med	–.07	–.04	–.03	No med
Activity	.26***	.09***	.17**	Part med	.28**	.08**	.20**	Part med	.25**	.07**	.18**	Part med
Neuroticism	–.19**	–.06***	–.13*	Part med	–.21**	–.10***	–.11	Full med	–.22***	–.08**	–.14**	Part med
Sensation seeking	.17**	.08**	.09	Full med	.14*	.03	.11	No med	.10	.04	.06	No med
SD3												
Narcissism	.28**	.12***	.16***	Part med	.33**	.16***	.17**	Part med	.32**	.11***	.20***	Part med
Psychopathy	–.01	–.05	.04	No med	–.27**	–.10**	–.17**	Part med	–.06	–.04	–.02	No med

*Note*: STE = Standardized total effect; SIE = Standardized indirect effect; SDE: Standardized direct effect; Full med = Full mediation; Part med = Partial mediation; No med = No mediation; * *p* < .05; ** *p* < .01; *** *p* < .001.

In model (b), significant indirect and direct effects of SD3’s narcissism on SPE were observed in each sample, suggesting a partial mediation of proactive attitude across cultural contexts. The indirect effects in this mediation were particularly strong in the three samples (Belgium, b = .12; Switzerland, b = .16; Togo, b = .11, all *p* < .001). Moreover, a partial mediation was observed with psychopathy in Switzerland with a strong indirect effect (b = –.10, *p* < .01), while no mediation was observed in the two other samples.

## Discussion

### Construct validity and measurement invariance across countries

The aim of this study was to investigate the relations between personality, the dark triad, proactive attitudes and SPE in three different contexts, while considering proactive attitude as a mediator and SPE as an outcome. Thus, we examined the cross-cultural invariance of each scale. We observed that all scales did reach metric invariance that allows comparisons of regression or mediation models across groups. The ZKA-PQ/SF and the PAS did not reach scalar invariance, so it is not relevant to compare mean scores across groups (Van de Vijver & Leung, 2001). In fact, it has been widely highlighted that scalar invariance is difficult to reach for most personality models (Rossier et al., 2016). However, the SD3, as well as the PES, exhibited scalar invariance across countries suggesting that means of these scales can be compared across countries. The ZKA-PQ/SF nearly reached metric invariance but not scalar, suggesting that context-specific norms have to be considered for this scale. Moreover, Aluja et al. (2019) demonstrated that this scale could easily attain metric invariance if its dimensions were considered separately.

### Contribution of normal and malevolent personality traits to self-perceived employability

Regarding personality, as hypothesized, aggressiveness (H1) was negatively related to SPE but only in the Swiss sample. This implies that in this country, low aggressive individuals reported higher levels of SPE than high aggressive individuals. One explanation for these differences could be that the reputation of Switzerland as a peaceful country might shape sensitivity to aggressiveness in this context in general compared to the two other ones, as the Swiss participants reported relatively higher aggressiveness. Describing one’s self relying on the group one belongs to, was referred to as reference-group effect, which is based on social comparison (Heine, Lehman, Peng, & Greenholtz, 2002). This might strengthen the perception of aggressiveness as deviant behavior that may not sustain the self-perception of employability in this context. If the reference-group effect could be a very plausible explanation here, very few studies have confirmed its impact on personality traits (Mõttus et al., 2012). As expected, neuroticism (H2) and activity (H3) were related negatively and positively, respectively, to SPE across the three countries, but not extraversion (H4). These findings imply that lower levels of neuroticism are associated with higher levels of SPE and that higher levels of activity are associated with higher levels of SPE across cultures, which is consistent with previous findings that considered the FFM (Wille et al., 2013). In fact, this study showed strong similarities with previous studies regarding the link between the above personality traits and other career outcomes, such as career engagement and work engagement (Nilforooshan & Salimi, 2016; Rossier et al., 2012). The contribution of sensation seeking (H5) was supported in our two European samples but not in our African sample, suggesting that this relation may be context-dependent. In fact, according to Hofstede’s (1991) classification, Belgium and Switzerland are members of the same pool of individualistic countries, which are culturally distant from Togo, which has been classified as a collectivist country. It is likely that the expression of sensation seeking in terms of behavior is moderated by contextual norms. For example, self-affirmation is more tolerated, even encouraged in individualistic cultures compared to collectivist ones. In fact, there is a difference between the willingness to sensation seek and its expression in terms of behavior, and it is very likely that the willingness to sensation seek is more easily expressed in individualistic cultures, where social pressure, hierarchy, power distance, and conformism are very important (Hofstede, 1991). This could explain why sensation seeking, which may be associated with self-affirmation (Churchill et al., 2018), failed to be related to SPE. Another explanation could be that sensation seeking is a composite of four distinct characteristics—adventure seeking, experience seeking, disinhibition and susceptibility/impulsivity— (Aluja et al., 2019), whose respective contributions to SPE are still unclear and need to be elucidated and clarified. Moreover, many individuals in collectivist countries grow up in relatively controlling families (valuing in-group regulation) characterized by the use of pressure and control rather than autonomy-supporting families (Shin & Kelly, 2013). It may follow that the link between activity and self-perception of employability is not direct as observed in our western samples. Instead, it may be mediated by other significant unexplored variables in these contexts. Furthermore, the rapid social, economic and technological changes and the recent economic crises have induced uncertainty, instability, increased competition and vulnerability in most western populations. Regarding careers, this situation henceforth requires individuals to update their competencies, flexibility, proactivity and a need to be active to escape the threat (Rossier, 2015). This could explain why higher levels of activity are significantly associated with higher levels of self-perceived employability in these contexts.

We expected positive associations between malevolent traits and SPE across countries (H6), which was partially confirmed. Results showed that narcissism significantly and positively predicted SPE across cultures. This suggested that high narcissistic individuals reported higher levels of SPE regardless of culture. This can be explained by the fact that narcissism is the most self-enhancement-oriented of the dark triad (Paulhus & Williams, 2002). Additionally, a recent investigation across 49 countries revealed that narcissism, unlike the Machiavellianism and psychopathy, was rated highly in 90% of cases (Jonason et al., 2020). Moreover, contrary to our expectation, psychopathy negatively related to SPE, but this relation was significant in the Swiss sample only. This finding seemed to highlight a sensitiveness of the swiss context to deficits in affect and self-control (i.e, psychopathy) in explaining subjective perceptions of employability. Further research should deepen this aspect. Finally, Machiavellianism did not predict SPE in any of the countries. One explanation can be that Machiavellians are reality-based (Paulhus and Williams, 2002); therefore, Machiavellianism seems to be more relevant in explaining operative career behaviors such as job performance or counterproductive work behaviors (O’Boyle et al., 2012) than nonoperative career variables such as SPE. Overall, this study supported the notion that, although correlated (positively) to a high extent, the toxic traits of narcissism, Machiavellianism, and psychopathy can have some specific (and even contrasted) contributions to career outcomes (Paulhus & Williams, 2002).

Findings also showed other cross-cultural differences: sex differences for self-perceived employability were observed in Togo (in favor of women) but not in Belgium or Switzerland. Although not part of our main hypotheses, this deserves a few comments. Atitsogbe, Moumoula, Rochat, Antonietti, and Rossier (2018) reported higher career indecision in Burkinabe women compared to men, compared to no gender differences among Swiss adults, suggesting that cultural conceptions of masculinity and femininity matters. Atitsogbe and colleagues (2018) argued that in most African societies, more responsibility is attributed to men in forming a family, which pushes them to make early career decisions. Drawing on these findings, one could expect differences in career outcomes such as perceived employability across these contexts. However, our findings showed higher perceived employability for women than for men in Togo. This could be attributed to gender equality campaigns that were strengthened in sub-Saharan Africa and implemented through law-making by policymakers in Togo in particular during the past decade. Togolese women have recently experienced a previously unprecedented rise to higher positions, which may have fostered self-confidence, self-efficacy and self-perceived employability among young generation women in this country.

### The mediation of proactive attitude between both normal and malevolent personality traits and perceived employability

The FFM framework has highlighted the indirect role of personality in the expression of behaviors through characteristic adaptation variables. In line with this theoretical position, we hypothesized that proactive attitude would mediate between personality dimensions and SPE (H7). We found that proactive attitude mediated between the personality dimensions of activity, neuroticism and narcissism and SPE across countries. Proactive attitude fully mediated between sensation seeking and SPE in the Belgian sample, and no such mediation was observed in either of the two other samples. Overall, the results suggested that one possibility for increasing SPE would be to encourage proactivity. The findings reaffirm the importance of proactivity in the process leading to career development outcomes (Ling et al., 2017).

### Limitations and further research

There are some limitations to this study. First, the study samples collected from each country were convenience samples and thus heterogeneous. Second, employment status was not assessed and subsequently not controlled for. Obtaining employment status and more homogenous samples would have provided more precision in the results. Third, the ethnocultural diversity, which may vary from one country to another, was not controlled for in this study. This may call into question the homogeneity of each national sample. Controlling for this parameter could have brought more precisions on our samples. This study contributed to the extension of the literature regarding the contribution of the AFFM and the SD3 to career development outcomes by adopting a cross-cultural approach. It supported the empirically significant contributions of the ZKA-PQ/SF’s activity and neuroticism measures and that of SD3’s narcissism to SPE in a cross-cultural manner. A significant contribution of the sensation seeking dimension was observed across the two European samples but failed in the African sample. Further research involving other cultural contexts would be necessary to elucidate the nature of the investigated relations and provide more substantial explanations regarding cross-cultural similarities and differences.

## Additional Files

The additional files for this article can be found as follows:

10.5334/pb.520.s1 Appendix A. French translation of the Short Dark Triad.

10.5334/pb.520.s2 Appendix B. French translation of the Proactive Attitude Scale.

## References

[1] Aluja, A., et al. (2019). Multicultural validation of the Zuckerman–Kuhlman–Aluja personality questionnaire shortened form (ZKA-PQ/SF) across 18 cultures. Assessment. Advanced online publication. DOI: 10.1177/1073191119831770 30880424

[2] Aluja, A., Kuhlman, M., & Zuckerman, M. (2010). Development of the Zuckerman–Kuhlman–Aluja personality questionnaire (ZKA-PQ): A factor/facet version of the Zuckerman–Kuhlman personality questionnaire (ZKPQ). Journal of Personality Assessment, 92, 416–431. DOI: 10.1080/00223891.2010.497406 20706928

[3] Atitsogbe, K. A., Mama, P. N., Sovet, L., Pari, P., & Rossier, J. (2019). Perceived employability and entrepreneurial intentions across university students and job seekers in Togo: The effect of career adaptability and self-efficacy. Frontiers in Psychology, 10, 1–14. DOI: 10.3389/fpsyg.2019.00180 30800087 PMC6376950

[4] Atitsogbe, K. A., Moumoula, I. A., Rochat, S, Antonietti, J.-P., & Rossier, J. (2018). Vocational interests and career indecision in Switzerland and Burkina Faso: Cross-cultural similarities and differences. Journal of Vocational Behavior, 107, 126–140. DOI: 10.1016/j.jvb.2018.04.002

[5] Blanch, A., Aluja, A., & Gallart, S. (2013). Personality assessment through internet: Factor analyses by age groups of the ZKA Personality Questionnaire. Psychologica Belgica, 53, 101–119. DOI: 10.5334/pb-53-4-101

[6] Byrne, B. M. (2010). Structural equation modeling with AMOS: Basic concepts, applications, and programming. New York, NY: Routledge.

[7] Chen, F. F. (2007). Sensitivity of goodness of fit indices to lack of measurement invariance. Structural Equation Modeling, 14, 464–504. DOI: 10.1080/10705510701301834

[8] Cheung, G. W., & Rensvold, R. B. (2002). Evaluating goodness-of-fit indexes for testing measurement invariance. Structural Equation Modeling: A Multidisciplinary Journal, 9, 233–255. DOI: 10.1207/S15328007SEM0902_5

[9] Churchill, S., Jessop, D. C., Goodwin, S., Ritchie, L., & Harris, P. R. (2018). Self-affirmation improves music performance among performers high on the impulsivity dimension of sensation seeking. Psychology of Music, 46, 292–302. DOI: 10.1177/0305735617705007

[10] Fugate, M., Kinicki, A. J., & Ashforth, B. E. (2004). Employability: A psycho-social construct, its dimensions, and applications. Journal of Vocational Behavior, 65, 14–38. DOI: 10.1016/j.jvb.2003.10.005

[11] Furnham, A., Richards, S. C., & Paulhus, D. L. (2013). The Dark Triad of personality: A 10-year review. Social and Personality Psychology Compass, 7, 199–216. DOI: 10.1111/spc3.12018

[12] Heine, S. J., Lehman, D. R., Peng, K., & Greenholtz, J. (2002). What’s wrong with cross-cultural comparisons of subjective Likert scales?: The reference-group effect. Journal of Personality and Social Psychology, 82(6), 903–918. DOI: 10.1037/0022-3514.82.6.903 12051579

[13] Hofstede, G. (1991). Empirical models of cultural differences In N. Bleichrodt, & P. J. D. Drenth (Eds.), Contemporary issues in cross-cultural psychology (pp. 4–20). Lisse, the Netherlands: Swets & Zeitlinger.

[14] Hofstede, G. (2001). Culture’s consequences: Comparing values, behaviors, institutions, and organizations across nations. Thousand Oaks, CA: Sage Publications.

[15] Hofstede, G., Hofstede, G. J., & Minkov, M. (2005). Cultures and organizations: Software of the mind (Vol. 2). New York: Mcgraw-hill.

[16] Hu, L., & Bentler, P. M. (1999). Cutoff criteria for fit indexes in covariance structure analysis: Conventional criteria versus new alternatives. Structural Equation Modeling: A Multidisciplinary Journal, 6, 1–55. DOI: 10.1080/10705519909540118

[17] Jonason, P., Żemojtel-Piotrowska, M., Piotrowski, J., Sedikides, C., Campbell, K. W., Gebauer, J., … Mamuti, A. (2020). Country-Level correlates of the Dark Triad traits in 49 countries. Journal of personality, 1–16. DOI: 10.1111/jopy.12569 32557617

[18] Jones, D. N., & Paulhus, D. L. (2014). Introducing the short dark triad (SD3): A brief measure of dark personality traits. Assessment, 21, 28–41. DOI: 10.1177/1073191113514105 24322012

[19] McAdams, D. P., & Pals, J. L. (2006). A new Big Five: Fundamental principles for an integrative science of personality. American Psychologist, 61, 204–217. DOI: 10.1037/0003-066X.61.3.204 16594837

[20] McCrae, R. R., & Costa, P. T. Jr. (1999). A five-factor theory of personality In L. Pervin & O. P. John (Eds.), Handbook of personality: Theory and research (2nd ed., pp. 139–153). New York, NY: Guilford Press.

[21] McHoskey, J. W., Worzel, W., & Szyarto, C. (1998). Machiavellianism and psychopathy. Journal of Personality and Social Psychology, 74, 192–210. DOI: 10.1037/0022-3514.74.1.192 9457782

[22] Mõttus, R., Allik, J., Realo, A., Pullmann, H., Rossier, J., Zecca, G., Ah-Kion, J., Amoussou-Yéyé, D., Bäckström, M., Barkauskiene, R., Barry, O., Bhowon, U., Björklund, F., Bochaver, A., Bochaver, K., de Bruin, G. P., Cabrera, H. F., Chen, S. X., Church, A. T., … Tseung, C. N. (2012). Comparability of self-reported conscientiousness across 21 countries. European Journal of Personality, 26(3), 303–317. DOI: 10.1002/per.840

[23] Nilforooshan, P., & Salimi, S. (2016). Career adaptability as a mediator between personality and career engagement. Journal of Vocational Behavior, 94, 1–10. DOI: 10.1016/j.jvb.2016.02.010

[24] O’Boyle, E. H., Jr., Forsyth, D. R., Banks, G. C., & McDaniel, M. A. (2012). A meta-analysis of the Dark Triad and work behavior: A social exchange perspective. Journal of Applied Psychology, 97, 557–579. DOI: 10.1037/a0025679 22023075

[25] Paulhus, D. L., & Williams, K. M. (2002). The dark triad of personality: Narcissism, Machiavellianism, and psychopathy. Journal of Research in Personality, 36, 556–563. DOI: 10.1016/S0092-6566(02)00505-6

[26] Potgieter, I., & Coetzee, M. (2013). Employability attributes and personality preferences of postgraduate business management students. SA Journal of Industrial Psychology, 39, 1–10. DOI: 10.4102/sajip.v39i1.1064

[27] Praskova, A., Creed, P. A., & Hood, M. (2015). Self-regulatory processes mediating between career calling and perceived employability and life satisfaction in emerging adults. Journal of Career Development, 42, 86–101. DOI: 10.1177/0894845314541517

[28] Razali, N. M., & Wah, Y. B. (2011). Power comparisons of Shapiro-Wilk, Kolmogorov-Smirnov, Lilliefors and Anderson-Darling tests. Journal of Statistical Modeling and Analytics, 2(1), 21–33.

[29] Riordan, C. M., & Vandenberg, R. J. (1994). A central question in cross-cultural research: Do employees of different cultures interpret work-related measures in an equivalent manner? Journal of Management, 20(3), 643–671. DOI: 10.1016/0149-2063(94)90007-8

[30] Rossier, J. (2015). Career adaptability and life designing In L. Nota & J. Rossier (Eds.), Handbook of life design: From practice to theory and from theory to practice (pp. 153–167). Göttingen, Germany: Hogrefe.

[31] Rossier, J., Aluja, A., Blanch, A., Barry, O., Hansenne, M., Carvalho, A. F., … Karagonlar, G. (2016). Cross-cultural generalizability of the alternative five-factor model using the Zuckerman-Kuhlman-Aluja personality questionnaire. European Journal of Personality, 30, 139–157. DOI: 10.1002/per.2045

[32] Rossier, J., Zecca, G., Stauffer, S. D., Maggiori, C., & Dauwalder, J. P. (2012). Career Adapt-Abilities Scale in a French-speaking Swiss sample: Psychometric properties and relationships to personality and work engagement. Journal of Vocational Behavior, 80, 734–743. DOI: 10.1016/j.jvb.2012.01.004

[33] Rothwell, A., Herbert, I., & Rothwell, F. (2008). Self-perceived employability: Construction and initial validation of a scale for university students. Journal of Vocational Behavior, 73, 1–12. DOI: 10.1016/j.jvb.2007.12.001

[34] Scherer, K. T., Baysinger, M., Zolynsky, D., & LeBreton, J. M. (2013). Predicting counterproductive work behaviors with sub-clinical psychopathy: Beyond the Five Factor Model of personality. Personality and Individual Differences, 55(3), 300–305. DOI: 10.1016/j.paid.2013.03.007

[35] Schmitz, G. S., & Schwarzer, R. (1999). Proaktive Einstellung von Lehrern: Konstruktbeschreibung und psychometrische Analysen. Zeitschrift für Empirische Pädagogik, 13(1), 3–27.

[36] Shapiro, S. S., & Wilk, M. B. (1965). An analysis of variance test for normality (complete samples). Biometrika, 52, 591–611. DOI: 10.2307/2333709

[37] Shrout, P. E., & Bolger, N. (2002). Mediation in experimental and nonexperimental studies: New procedures and recommendations. Psychological Methods, 7, 422–445. DOI: 10.1037/1082-989X.7.4.422 12530702

[38] Thomas, J. P., Whitman, D. S., & Viswesvaran, C. (2010). Employee proactivity in organizations: A comparative meta-analysis of emergent proactive constructs. Journal of Occupational and Organizational Psychology, 83(2), 275–300. DOI: 10.1348/096317910X502359

[39] Thompson, B., & Melancon, J. G. (1996). Using item ‘testlets’/‘parcels’ in confirmatory factor analysis: An example using the PPSDQ-78. Paper presented at the annual meeting of the Mid-South Educational Research Association, Tuscaloosa, AL.

[40] Tokar, D. M., Fischer, A. R., & Subich, L. M. (1998). Personality and vocational behavior: A selective review of the literature, 1993–1997. Journal of Vocational Behavior, 53, 115–153. DOI: 10.1006/jvbe.1998.1660

[41] Udayar, S., Fiori, M., Thalmayer, A. G., & Rossier, J. (2018). Investigating the link between trait emotional intelligence, career indecision, and self-perceived employability: The role of career adaptability. Personality and Individual Differences, 135, 7–12. DOI: 10.1016/j.paid.2018.06.046

[42] Van de Vijver, F., & Leung, K. (2001). Personality in cultural context: Methodological issues. Journal of Personality, 69, 1007–1031. DOI: 10.1111/1467-6494.696173 11767816

[43] Van Hootegem, A., De Witte, H., De Cuyper, N., & Elst, T. V. (2018). Job insecurity and the willingness to undertake training: The moderating role of perceived employability. Journal of Career Development. DOI: 10.1177/0894845318763893

[44] Wille, B., De Fruyt, F., & Feys, M. (2013). Big five traits and intrinsic success in the new career era: A 15-Year longitudinal study on employability and Work–Family conflict. Applied Psychology, 62, 124–156. DOI: 10.1111/j.1464-0597.2012.00516.x

[45] Zecca, G., Györkös, C., Becker, J., Massoudi, K., de Bruin, G. P., & Rossier, J. (2015). Validation of the French Utrecht Work Engagement Scale and its relationship with personality traits and impulsivity. Revue Européenne de Psychologie Appliquée/European Review of Applied Psychology, 65, 19–28. DOI: 10.1016/j.erap.2014.10.003

