# Supplementary material for: Normal Personality, the Dark Triad, Proactive Attitude and Perceived Employability: A Cross-Cultural Study in Belgium, Switzerland and Togo
Source: Psychol Belg. 2020 Jul 22;60(1):217–35. doi: 10.5334/pb.520 (PMC7380056; doi:10.5334/pb.520)
Supplement: Appendix A. — French translation of the Short Dark Triad. [file pb-60-1-520-s1.pdf]

Appendix A

French translation of the Short Dark Triad.

Veuillez indiquer dans quelle mesure vous êtes en accord ou en désaccord avec les propositions suivantes.

1	2	3	4	5
fortement en désaccord	en désaccord	ni en accord ni en désaccord	en accord	fortement en accord

Machiavellianism

1. It's not wise to tell your secrets / Il n'est pas prudent de révéler ses secrets.
2. I like to use clever manipulation to get my way / J'aime manipuler les gens de façon habile pour obtenir ce que je veux.
3. Whatever it takes, you must get the important people on your side / Il faut tout faire pour mettre les gens influents de son côté.
4. Avoid direct conflict with others because they may be useful in the future / C'est bien d'éviter d'être en conflit direct avec les autres parce qu'ils pourraient être utiles dans le futur.
5. It's wise to keep track of information that you can use against people later / Il est sage de conserver les informations qu'on peut utiliser contre les gens par la suite.
6. You should wait for the right time to get back at people / Il est préférable d'attendre le bon moment pour se venger.
7. There are things you should hide from other people because they don't need to know / Il vaut mieux cacher aux autres les choses qu'ils n'ont pas besoin de savoir.
8. Make sure your plans benefit you, not others / Je m'assure que mes projets ne bénéficient qu'à moi, et non aux autres.
9. Most people can be manipulated / La plupart des gens peuvent être manipulés.

Narcissism

1. People see me as a natural leader / Les gens trouvent que je suis naturellement un-e leader.
2. I hate being the center of attention / Je déteste être au centre de l'attention. (R)
3. Many group activities tend to be dull without me / Beaucoup d'activités de groupe ont tendance à être ennuyeuses sans moi.
4. I know that I am special because everyone keeps telling me so / Je sais que je suis spécial-e car les gens n'arrêtent pas de me le dire.
5. I like to get acquainted with important people / J'aime fréquenter des gens importants.
6. I feel embarrassed if someone compliments me / Je suis gêné-e si quelqu'un me fait des compliments.
7. I have been compared to famous people / On m'a déjà comparé-e à des gens célèbres. (R)
8. I am an average person / Je suis une personne ordinaire. (R)
9. I insist on getting the respect I deserve / Je tiens à obtenir le respect que je mérite.

Psychopathy

1. I like to get revenge on authorities / J'aime me venger des autorités.
2. I avoid dangerous situations / J'évite les situations dangereuses. (R)
3. Payback needs to be quick and nasty / Une vengeance doit être rapide et méchante.
4. People often say I'm out of control / Les gens disent souvent que je suis incontrôlable.
5. It's true that I can be mean to others / Il est vrai que je peux être méchant-e avec les autres.
6. People who mess with me always regret it / Les gens qui me cherchent des ennuis finissent toujours par le regretter.
7. I have never gotten into trouble with the law / Je n'ai jamais eu de problèmes avec la justice. (R)
8. I enjoy having sex with people I hardly know J'aime avoir des rapports sexuels avec des gens que je connais à peine.
9. I'll say anything to get what I want / Je suis capable de dire n'importe quoi pour obtenir ce que je veux.
