# Supplementary material for: Normal Personality, the Dark Triad, Proactive Attitude and Perceived Employability: A Cross-Cultural Study in Belgium, Switzerland and Togo
Source: Psychol Belg. 2020 Jul 22;60(1):217–35. doi: 10.5334/pb.520 (PMC7380056; doi:10.5334/pb.520)
Supplement: Appendix B. — French translation of the Proactive Attitude Scale. [file pb-60-1-520-s2.pdf]

Appendix B

French translation of the Proactive Attitude Scale items

1. I spend time identifying long-range goals for myself / Je consacre du temps à me définir des objectifs à long terme.
2. I feel in charge of making things happen / Je me sens responsable de faire en sorte que les choses se réalisent.
3. I feel responsible for my own life / Je me sens responsable de ma vie.
4. I feel driven by my personal values / Je me sens guidé par mes valeurs personnelles.
5. I am driven by a sense of purpose / Je suis poussé par le sentiment d'avoir un but dans la vie.
6. I am able to choose my own actions / Je suis capable de choisir mes actions.
7. I focus my efforts on things that I can control / Je concentre mes efforts sur les choses que je peux contrôler.
8. There are abundant opportunities that await me / De nombreuses opportunités m'attendent.
